# The HOPE for Pandora’s Box of artificial circular RNA immunogenicity

**DOI:** 10.1016/j.omtn.2022.04.029

**Published:** 2022-05-13

**Authors:** Faryal Mehwish Awan

**Affiliations:** 1 Department of Medical Lab Technology, The University of Haripur (UOH), Haripur, 22620 Khyber Pakhtunkhwa, Pakistan

A recent paper by Breuer et al. in *Molecular Therapy Nucleic Acids* has addressed a major question of whether artificial circular RNAs (circRNAs) escape immune-surveillance or not.^1^ The authors revealed that the immunogenic potential of circRNAs (developed to sequester an oncogenic miRNA) is strongly affected by the purification strategy where completely purified circRNAs appeared relatively less immunogenic compared with unpurified or incompletely purified ones. In order to study the innate immune responses triggered by artificial circRNAs, the authors designed an artificial circRNA sponge (approximately 200 nt in length with a double-stranded 11 nt stem loop) and an extensively double-stranded circRNA (circRNA-ds) containing a randomized self-complementary sequence of 50 bp. The authors utilized T4 RNA ligase I for *in vitro* circularization. Transcriptome analysis was performed after transfecting A549 cells with circRNAs in order to examine innate immune system activation. Findings revealed minimal innate immunity activation (observed only after treatment with high doses of the circRNAs) as well as no transcriptional up-regulation of RNA sensors. On the contrary, circRNAs-ds not only up-regulate genes involved in innate immune responses but also up-regulate PKR, a key RNA sensor. The authors proposed that factors like secondary-structure elements, sequence composition, or liposome-based transfection may play a role in the induction of the innate immune response apart from contaminants. For the exclusion of contaminants of transcription reaction, stringent purification was performed via gel extraction. The authors concluded that a polyacrylamide-urea gel purification approach appears to be the most efficient method for the purification of circRNAs. *In vitro* synthesis of immunogenicity-free circRNAs have potential clinical relevance and biomedical applications. This study will make a significant contribution in the field of RNA therapeutics by providing the scientific community with a strategy for reducing the immunogenicity of produced RNA circles. One of the important points that needs to be addressed here is whether the same phenomena will be observed for longer circRNAs, as T4 RNA ligase has limited circularization capacity when the RNA is longer.^2^

**Figure 1:**
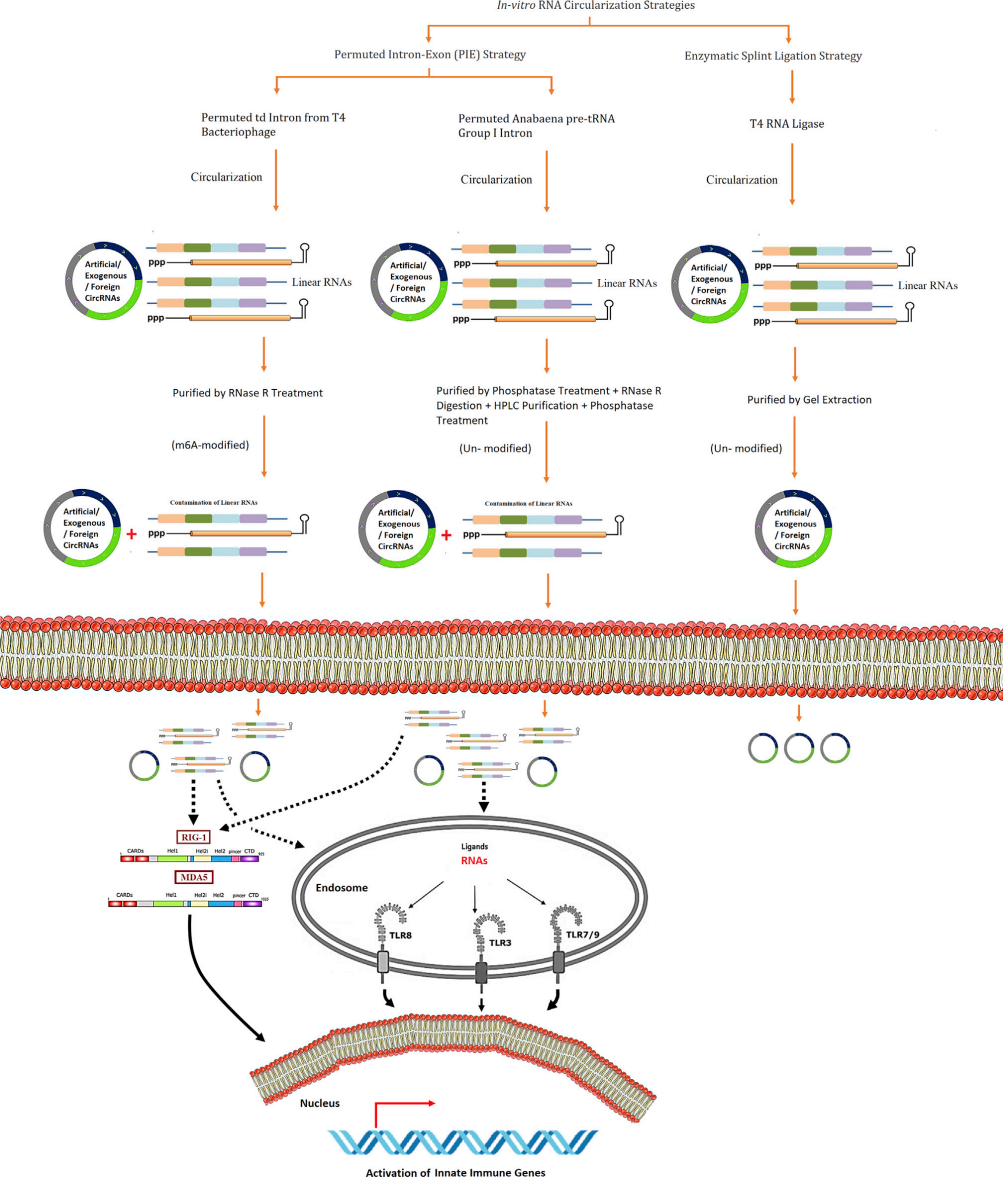
Immune recognition of artificial circRNAs synthesized and purified by different approaches

Several studies have investigated the potential immunogenicity of circRNAs relative to linear RNAs and have reached varying conclusions (Figure 1). Findings of all these studies revealed that RNA-sensor-mediated immune activation of artificial circRNAs is highly dependent on a number of factors including length, construct design, production, purification strategies, and modifications, as well as the dose of the circRNA administered. Work done by Liu et al. revealed similar findings to Breuer et al.^2^ The authors revealed that circRNAs synthesized by permuted td introns from T4 bacteriophage or by pre-tRNA group I introns could induce an immune response because of the extraneous fragments that can distort circRNAs folding. In contrast, circRNAs synthesized by T4 RNA ligase (containing short double-stranded RNA [dsRNA] regions) without extraneous fragments exhibited minimal immunogenicity. The authors performed purification of circRNAs via denaturing PAGE and RNase R treatments. In another study, transfection of artificially produced circRNA by Chen et al. led to a robust induction of cytokines in an RIG-I-dependent manner.^3^ The authors utilized RNase R treatment for purification of circRNAs. Later on, Wesselhoeft et al. further investigated this question and concluded that contaminating RNA species elicited innate immune responses in RNase R-purified circRNAs.^4^ In order to remove contaminants, the authors performed additional steps for circRNA purification using phosphatase treatment and RNase R digestion, along with high-performance liquid chromatography (HPLC) purification, and found that those circRNAs purified using this strategy do not elicit substantial innate immune responses. These results demonstrated that circRNA purity strongly affects its immunogenic potential. The authors further examined and investigated the effect of m1ψ modification on purified circRNAs and concluded that nucleoside modification of circRNAs is not necessary for protection against innate immune sensors. Given these inconsistencies in the results, Chen et al. reexamined the immunogenicity of circRNAs.^5^ The authors found that artificially produced circRNAs that had been subjected to further purification, mirroring the work by Wesselhoeft et al., are still immunogenic.

Findings of all these studies are debatable, which further highlights the fact that several additional factors could impact the observed immunogenicity of artificial circRNAs apart from contaminating RNA species, e.g., the study design of Chen et al. included synthesis of circRNAs with permuted group I introns from phage T4 td gene, circRNAs approximately 1500 nt in length, modifications done (none), cell types used for transfection (HeLa, HEK293T, HaCaT, RAW 264.7, and mouse embryonic fibroblast [MEF]), transfection reagent used (Lipofectamine 2000), amount of circRNA transfected (500 ng), and RNase R treatment used to purify circRNAs;^3^ the study design of Chen et al. included production of circRNAs with permuted group I introns from phage T4 td gene, circRNAs approximately 1,500 nt in length, modifications done (N6-methyladenosine), cell types used for transfection (HeLa and HEK293T), transfection reagent used (Lipofectamine 3000), amount of circRNA transfected (500 ng), RNase R enrichment and phosphatase treatment to purify circRNAs, dose of circRNA administered (25 μg), and route of inoculation (subcutaneous);^5^ the study design of Wesselhoeft et al. included production of circRNAs with permuted *Anabaena* pre-tRNA group I introns, circRNAs approximately 1,200 nt in length, modifications done (m1ψ), cell types used for transfection (HEK293, A549, HeLa, and RAW264.7), transfection reagent used (Lipofectamine MessengerMAX), amount of circRNA transfected (40–200 ng), phosphatase treatment, RNase R digestion, and HPLC to purify circRNAs, dose of circRNA (350 ng), and route of inoculation (visceral fat);^4^ the study design of Liu et al. included production of circRNAs with permuted group I introns from phage T4 td gene, *Anabaena* pre-tRNA group I introns as well as with T4 RNA ligase, circRNAs approximately 336, 410, and 522 nt in length, modifications done (none), cell types used for transfection (A549, HeLa, HEK293, and 293FT), transfection reagent used (Lipofectamine MessengerMAX reagent), amount of circRNA transfected (200 ng), and denaturing PAGE to purify circRNAs;^2^ and the study design of Breuer et al. included production of circRNAs with T4 RNA ligase I, circRNAs approximately 200 nt in length, modifications done (none), A549 cells were used for transfection, transfection reagent used (Lipofectamine 2000), amount of circRNA transfected (250 ng), and polyacrylamide-urea gel extraction to purify circRNAs.^1^ In one of the recent papers, the authors reported differences in circRNA migration behavior dependent on the agarose gel systems used.^6^ The E-Gel EX electrophoresis system was utilized by Wesselhoeft et al. to distinguish circRNAs from other RNA species, which is different from the traditional agarose systems used in other studies. The authors suggested that various factors can significantly impact the behavior of circRNA migration in different gel systems, e.g., sample buffer reagents used, circRNA length, and type of RNA modifications done. Keeping all these differences among studies—cell types used, transfection reagents, the amount of circRNAs transfected, length of circRNAs, modifications done, and the dose, as well as the route of inoculation—could contribute to differential activation of innate pathways. All these factors should be further evaluated to reconcile discordant results regarding the immunogenic potential of *in vitro* circularized RNAs.

In summary, it is still difficult to make definitive conclusions about circRNA immunogenicity, and there are still several unanswered questions that need to be addressed before reaching a final conclusion, e.g., does only purification strategy matte in evading RIG-I detection? What is the role of differences in secondary structures, tertiary structures, and sequence length in the immunogenic potential of circRNAs? How could the extra fragments of pre-tRNA and td genes impact the immunogenic potential of circRNAs? Keeping all the work done on the immunogenic potential of artificial circRNAs and in order to answer all of the aforementioned questions, further in-depth investigations of cellular immune responses to circRNAs with varying lengths and shapes produced by different approaches in different cell lines and *in vivo* should be tested and are therefore still required.
